# Study of the Magnesium Comenate Structure, Its Neuroprotective and Stress-Protective Activity

**DOI:** 10.3390/ijms24098046

**Published:** 2023-04-28

**Authors:** Stanislav Kozin, Alexandr Kravtsov, Lev Ivashchenko, Victor Dotsenko, Lada Vasilyeva, Alexander Vasilyev, Elena Tekutskaya, Nicolai Aksenov, Mikhail Baryshev, Anna Dorohova, Lilia Fedulova, Stepan Dzhimak

**Affiliations:** 1 Physics and Technology Faculty, Kuban State University, 350040 Krasnodar, Russia; 2 Laboratory of Problems of Stable Isotope Spreading in Living Systems, Federal Research Center the Southern Scientific Center, Russian Academy of Sciences, 344006 Rostov-on-Don, Russia; 3 Laboratory of Technologies for the Production of Physiologically Active Substances, Kuban State Technological University, 350072 Krasnodar, Russia; 4 Faculty of Chemistry and High Technologies, Kuban State University, 350040 Krasnodar, Russia; 5 Faculty of Chemistry and Pharmacy, North Caucasus Federal University, 355017 Stavropol, Russia; 6 Experimental Clinic-Laboratory of Biologically Active Substances of Animal Origin, The V. M. Gorbatov Federal Research Center for Food Systems, Russian Academy of Sciences, 109316 Moscow, Russia

**Keywords:** comenic acid, magnesium ions, magnesium complex compounds, immobilization stress, antioxidants, neuroprotectors, neuritic growth, excitotoxicity

## Abstract

The crystal structure and the biological activity of a new coordination compound of magnesium ions with comenic acid, magnesium comenate, was characterized and studied. Quantitative and qualitative analysis of the compound was investigated in detail using elemental X-ray fluorescent analysis, thermal analysis, IR-Fourier spectrometry, UV spectroscopy, NMR spectroscopy, and X-ray diffraction analysis. Based on experimental analytical data, the empirical formula of magnesium comenate [Mg(HCom)_2_(H_2_O)_6_]·2H_2_O was established. This complex compound crystallizes with eight water molecules, six of which are the hydration shell of the Mg^2+^ cation, and two more molecules bind the [Mg(H_2_O)_6_]^2+^ aquacation with ionized ligand molecules by intermolecular hydrogen bonds. The packing of molecules in the crystal lattice is stabilized by a branched system of hydrogen bonds with the participation of solvate water molecules and oxygen atoms of various functional groups of ionized ligand molecules. With regard to the biological activity of magnesium comenate, a neuroprotective, stress-protective, and antioxidant effect was established in in vitro and in vivo models. In in vitro experiments, magnesium comenate protected cerebellar neurons from the toxic effects of glutamate and contributed to the preservation of neurite growth parameters under oxidative stress caused by hydrogen peroxide. In animal studies, magnesium comenate had a stress-protective and antioxidant effect in models of immobilization–cold stress. Oral administration of magnesium comenate at a dose of 2 mg/kg of animal body weight for 3 days before stress exposure and for 3 days during the stress period led to a decrease in oxidative damage and normalization of the antioxidant system of brain tissues against the background of induced stress. The obtained results indicate the advisability of further studies of magnesium comenate as a compound potentially applicable in medicine for the pharmacological correction of conditions associated with oxidative and excitotoxic damage to nerve cells.

## 1. Introduction

Due to the progressive aging of the population and environmental pollution with pesticides and heavy metals, the number of people with chronic neurodegeneration is increasing every year [1]. In absolute terms, it is estimated that around 36 million people worldwide had dementia in 2010, around 47 million in 2015, and by 2050, this number is expected to reach 115–130 million [1,2]. In addition, chronic neurodegeneration can be a consequence of traumatic brain injury [3]. More than 50 million new cases of traumatic brain injury are registered annually in the world, of which more than 90% are of mild severity. However, mild traumatic brain injury is dangerous because of long-term symptoms that can lead to the development of neurodegenerative processes.

The brain is the most sensitive organ to the disturbance of redox homeostasis [4,5,6,7]. This is due to the following factors. The brain consumes the largest amount of oxygen and glucose (20% and 25%, respectively). Auto-oxidation of neurotransmitters occurs in the brain. Brain tissues have moderate endogenous antioxidant protection, which is expressed by low catalase activity and low levels of reduced glutathione. There is a relatively high amount of polyunsaturated fatty acids and transition metals present in the brain tissues. In this regard, oxidative stress (OS) is one of the leading links underlying the death and damage of brain cells. The use of antioxidants in modeling the neurodegenerative diseases in animals showed high efficiency, but at the stages of clinical trials, it turned out to be ineffective [8]. Therefore, the search for new molecules with antioxidant and neuroprotective properties for the prevention and treatment of neurodegenerative diseases remains relevant.

Magnesium ions perform a number of protective functions in the brain. The protective effect of magnesium ions is caused by the ability to inhibit N-methyl-D-aspartate NMDA glutamate receptors, thereby preventing sustained stimulation of these receptors, which leads to neuronal death [9]. Ca^2+^ homeostasis in brain neurons is also controlled by magnesium ions [10]. Mg^2+^ can block mitochondrial opening and prevent apoptosis [11]. Magnesium ions protect the integrity and function of the blood–brain barrier [12]. Low magnesium levels increase tachykinin peptide, which activates microglia and releases pro-inflammatory mediators and nitric oxide [13]. Thus, physiologically normal magnesium concentrations protect brain cells from oxidative stress and neuroinflammation. The state of acute and chronic stress leads to the depletion of intracellular Mg^2+^ and its loss in the urine due to the release of a large amount of adrenaline and norepinephrine, which, in turn, contributes to the removal of magnesium ions from the cells [14]. In addition, magnesium ions are agonists of gamma-aminobutyric acid receptors (GABA_A_ receptors), which are involved in the development of the anxiolytic effect [15].

Magnesium drugs are considered as neuroprotective agents due to their high biological activity. Magnesium ions support cognitive function and synaptic plasticity in sporadic Alzheimer’s model rats in animal experiments [16]. In clinical practice, magnesium sulfate is used for fetal neuroprotection [17]. There are no clinical data definitively proving the benefit of magnesium sulfate in stroke patients, but the use of magnesium and potassium salts by patients for six months improved clinical indicators [18].

An urgent task of pharmacology is the search for new ligands that would enhance the effect of magnesium ions due to synergy. The established protective effect of comenic acid gives grounds to consider it as a potential ligand for Mg^2+^ ions in the context of the search for new neuroprotective agents. For comenic and meconic acids, cytoprotective activity was established under the excitotoxic effect of glutamate, as well as a high antioxidant potential in model systems [19,20,21]. Comenic acid is an active component of the drug Baliz-2, used in the treatment of peptic ulcers of the gastrointestinal tract and burns of skin surfaces. It is of note that the drug has practically no side effects [22,23]. Comenic acid has a wide range of biological activity. In addition to these properties, it has a neurotrophic effect, prevents an increase in long-term potentiation under stress, and has anti-amnesic and anxiolytic effects [21,24,25,26,27].

The purpose of this work was to study the structure of the complex compound of magnesium with comenic acid (magnesium comenate—MC) and to study the antioxidant and neuroprotective properties of this compound.

## 2. Results

### 2.1. Chemical Part

#### 2.1.1. IR Spectroscopy

**Figure 1:**
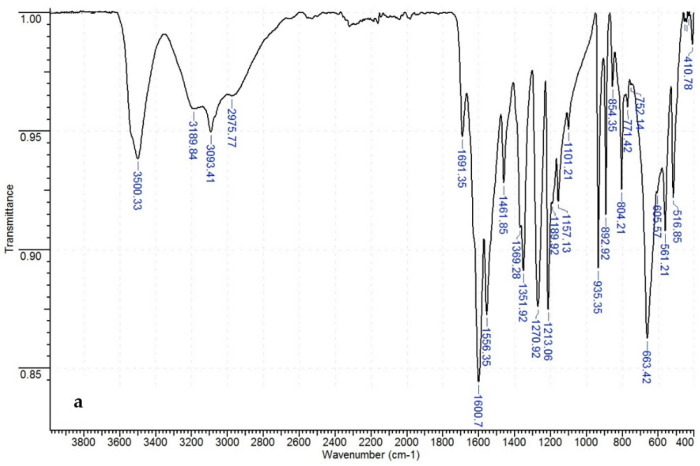

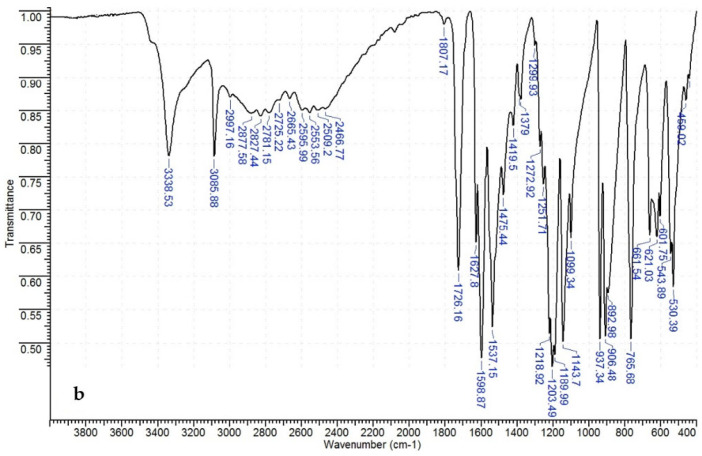
Infrared spectra: (**a**) comenic acid; (**b**) complex compound [Mg(Hcom)_2_(H_2_O)_6_]·2H_2_O.

The mode of coordination of comenic acid anions in the complex with Mg^2+^ was determined from the data of IR spectroscopy. The assignment of the characteristic absorption bands in the IR spectrum (Figure 1) was carried out by comparing it with the spectrum of comenic acid (Figure 1) and analyzing the literature data on β-hydroxy-γ-pyrones. In the IR spectrum, there is a shift of 162 cm^−1^ of the ν_O–H_ band in the composition of comenic acid (3500 cm^−1^), which indicates the breaking of intramolecular hydrogen bonds in the initial ligand dimers (absorption bands in the region of 3000–2400 cm^−1^). A broadening of the band near ν_C–H_ of the γ-pyrone structure is observed, which overlaps with ν_O–H_ (H_2_O). This is confirmed by thermal analysis data, which show the presence of a large number of coordinated water molecules in the complex. In the IR spectrum of the complex, the band of stretching vibrations of the COOH group splits into ν_as_(COO^–^) 1601 cm^−1^ and ν_sy_(COO^–^) 1352 cm^−1^. The difference is 249 cm^−1^, which is much more than 200 cm^−1^ and, therefore, in the complex, each ligand binds independently. The presence of the Mg–O bond is confirmed by the appearance of a medium-intensity absorption band at 517 cm^−1^.

#### 2.1.2. Thermal Analysis

**Figure 2:**
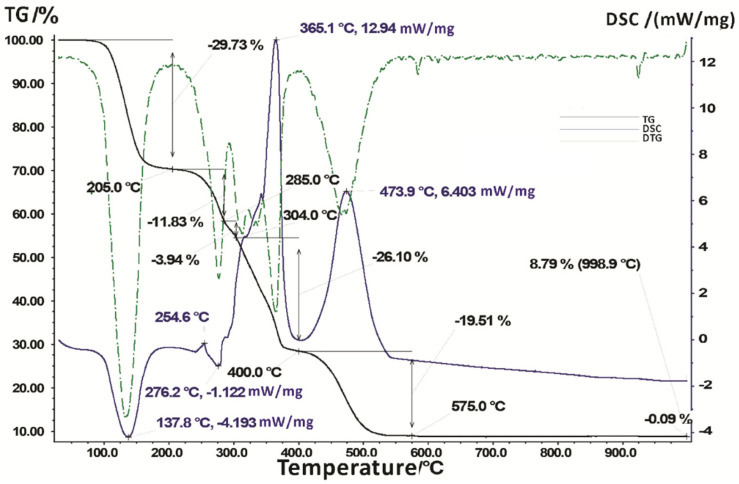
TG, DTG, DSC curves of a complex compound [Mg(Hcom)_2_(H_2_O)_6_]·2H_2_O.

The thermal behavior and hydrate composition of the coordination compound were studied using thermogravimetric analysis data. Figure 2 shows the curves of the synchronous thermal analysis of the magnesium comenate.

The thermolysis of this compound includes several stages (Table 1). The differential scanning calorimetry (DSC) curve shows an intense endo effect (−4.193 mW/mg), which indicates that at temperatures from 25 up to 205 °C, dehydration occurs with a weight loss of 29.73% according to the thermogravimetric curve, which corresponds to a detachment of eight water molecules.

This conclusion is in good agreement with the literature data. Thus, during the thermal decomposition of a complex of magnesium with nicotinic acid, an endoeffect is observed at 130–200 °C, which indicates a stepwise dehydration of water molecules unequally bound to the central ion [28]. Thermal analysis of the complex of magnesium with tripeptide glycine shows that at a temperature of 191.5 °C, there is a loss of two molecules of coordination water [29]. A further increase in temperature, in the range of 205–304 °C, is accompanied by a weak absorption of heat (–1.122 mW/mg) with a weight loss of 11.83 and 3.94%, which confirms the processes of intramolecular dehydration (detachment of one OH group) and decarboxylation of ligand molecules, surrounding the Mg^2+^ cation. It can be assumed that complex transformations occur in this case due to intramolecular rearrangement into a more favorable thermodynamic state. In the temperature ranges of 304–400 and 400–575 °C, the DSC curve shows two strongly pronounced exo-effects (365.1 °C, 12.94 mW/mg and 473.9 °C, 6.403 mW/mg, respectively) with burn-up proceeding in two distinct stages, the remaining part of two molecules of comenic acid with a decrease in mass by 26.10%, and then by 19.51%. The end product of thermolysis is magnesium oxide, which is confirmed by the result of X-ray fluorescent elemental analysis of the isolated ashing product. Table 2 presents the generalized results of the physicochemical research methods used to find the general formula of magnesium comenate—[Mg(Hcom)_2_(H_2_O)_6_]·2H_2_O.

#### 2.1.3. X-ray Diffraction Analysis

Triclinic crystals suitable for X-ray analysis were obtained by recrystallization of magnesium comenate from an aqueous solution. Several single crystals were obtained, the size of which were suitable for X-ray diffraction studies (photos of obtained crystals in Supplementary Figure S1). Structures were solved using the SHELXT program [30]. The remaining non-hydrogen atoms were localized by a direct method by successive calculations of difference Fourier maps. Atomic positions were refined by full-matrix least squares using F^2^*_hkl_* in the anisotropic approximation for all non-hydrogen atoms using SHELXL [30]. The contributions of hydrogen atoms are taken into account in the calculations but are not refined. In all cases, the locations of the largest peaks, as well as the values of the residual electron density in the final difference Fourier maps, are chemically insignificant.

**Figure 3:**
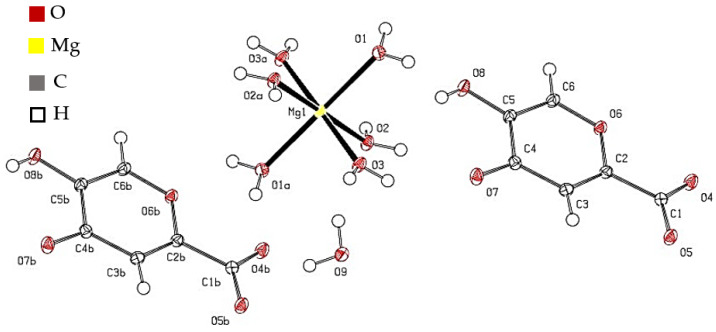
General view of a molecule of magnesium complex compound with 5-hydroxy-4-oxo-4*H*-pyran-2-carboxylic acid in a crystal.

**Figure 4:**
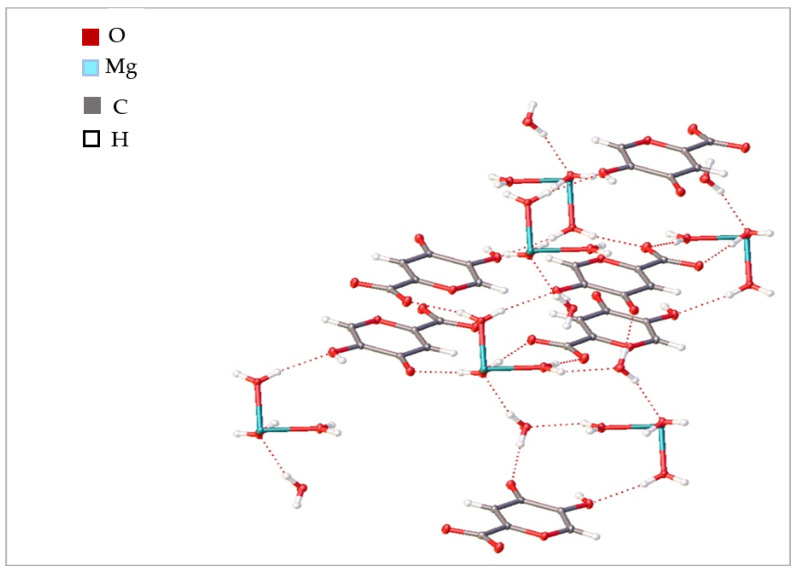
Short intermolecular contacts.

The complex compound crystallizes with eight water molecules, with six molecules being the hydration shell of the Mg^2+^ cation, and two more molecules bending the [Mg(H_2_O)_6_]^2+^ aquacation with ionized ligand molecules by intermolecular hydrogen bonds. The molecular structure of [Mg(HCom)_2_(H_2_O)_6_]·2H_2_O is shown in Figure 3; dashed lines show shortened contacts (Figure 4) that are observed in this structure. The packing of molecules in the crystal lattice is stabilized by a branched system of hydrogen bonds with the participation of solvate water molecules and oxygen atoms of various functional groups of ionized ligand molecules.

The main crystallographic characteristics and parameters of X-ray diffraction experiments for the compound are given in Supplementary (Table S1). The most important bond lengths and bond angles within the coordination polyhedron are presented in Supplementary (Tables S2 and S3). The Mg^2+^ ion is in a six-coordinated oxygen environment formed by intrasphere-coordinated water molecules.

The atomic coordinates and other structural parameters were deposited at the Cambridge crystallographic data center (CCDC no. 2207835); www.ccdc.cam.ac.uk/structures, accessed on 21 November 2022).

### 2.2. Biological Part

#### 2.2.1. Antioxidant Activity in the “Citrate-Phosphate-Luminol” CPL Model System

The results of the study of the antioxidant properties of magnesium comenate in vitro in the CPL model system (Table 3) show a significant decrease in the content of free radicals in comparison with the control. At the same time, in terms of efficiency, magnesium comenate practically does not differ from comenic acid, and similarly changes in concentration dependence. Thus, an increase in the concentration of both comenic acid and magnesium comenate in the test solution from 0.01 mg/mL to 0.1 mg/mL contributes to a significant (by ≈36%) increase in the level of free radical quenching.

Thus, magnesium comenate has a pronounced antioxidant effect, while its antioxidant properties practically do not differ from the antioxidant properties of comenic acid.

#### 2.2.2. Neurotrophic Action in OS

**Figure 5:**
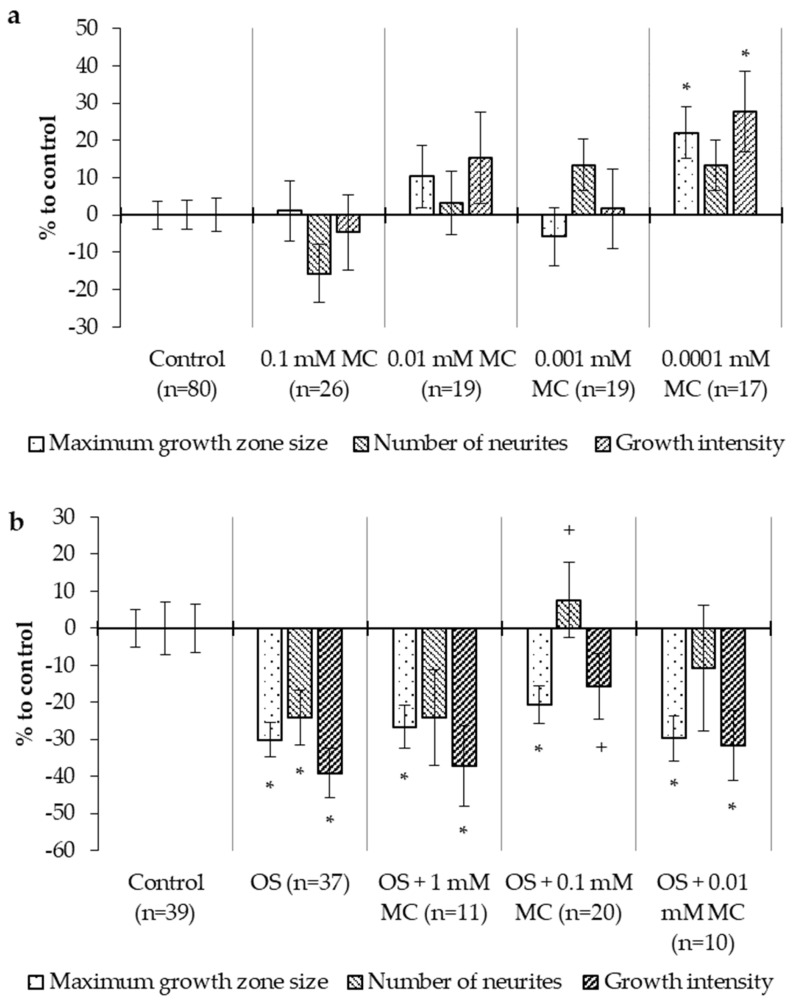
Effect of magnesium comenate on the growth of neurites in the spinal ganglia of chickens under normal conditions (**a**) and oxidative stress (**b**) (OS). *—*p* < 0.05 in relation to control, +—*p* < 0.05 in relation to oxidative stress.

As the analysis of the data under normal conditions shows, exposure to MC at a concentration of 0.0001 mM leads to the stimulation of maximum growth zone size (MGZS) and growth intensity (GI) by 22% and 28%, respectively (Figure 5). At concentrations from 1 mM to 0.001 mM, MC has no significant effect on the growth of spinal ganglia neurites under normal conditions. Exposure to hydrogen peroxide (Figure 5) leads to the inhibition of neurite growth: MGZS decreases by 30%, the number of neurites (NN) by 24%, and GI by 39% (*p* < 0.05). Magnesium comenate at a concentration of 0.1 mM has a protective effect, largely neutralizing the consequences of oxidative stress: the values of NN and GI significantly exceed those in comparison of the OS group. In relation to the control, the change in parameters is +8% for NN and −16% for GI and is not statistically significant. The MGZS value is lower than in control cultures by 21% (*p* < 0.05).

#### 2.2.3. Neuroprotective Action during Excitotoxic Exposure

**Figure 6:**
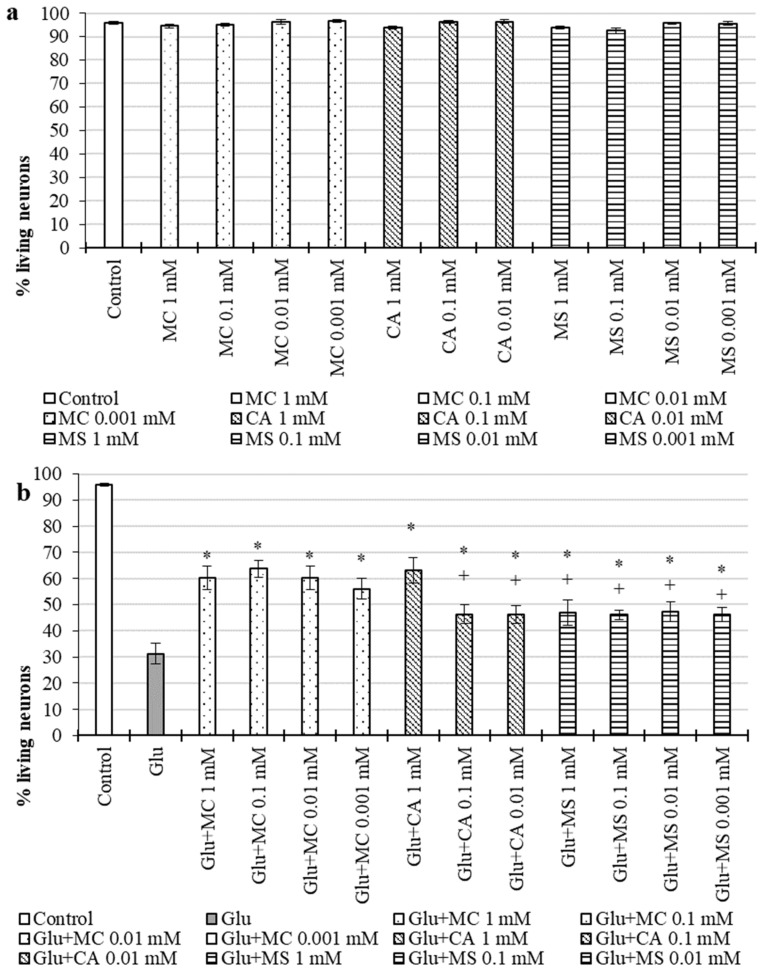
Effect of magnesium comenate, comenic acid (CA) and magnesium sulfate (MS) on the survival of cultured rat cerebellar neurons: (**a**) under normal conditions and (**b**) under excitotoxic effects of glutamate (Glu). *—*p* < 0.05 in relation to the control, +—*p* < 0.05 in relation to the point “Glu + MC 0.1 mM”.

The results of the study of neuroprotective activity under excitotoxic exposure show that, under normal conditions (Figure 6), the number of surviving neurons under the influence of comenate magnesium, comenic acid, and magnesium sulfate, regardless of their concentration, practically do not differ from the control value of 96% of neurons.

After the treatment of cultures with glutamate (Figure 6), a sharp decrease in the survival of neurons is observed (up to 31.2%). The use of the studied substances after exposure to glutamate in all the studied concentrations leads to a significant increase in the proportion of living neurons. However, there are also significant differences in efficiency. The introduction of MC into cultures treated with glutamate at concentrations of 1, 0.1, 0.01, and 0.001 mM increases the survival of neurons to 60, 64, 60, and 56%, respectively. That is, all concentrations show approximately equal efficiency. Comenic acid shows a different effect. At a concentration of 1 mM, the neuroprotective effect of comenic acid is not inferior to that of magnesium—63% of neurons remain intact, while at 0.1 and 0.01 mM the effect decreases by about half: 46% of neurons remain alive with both contractions. Magnesium sulfate shows an approximately equal neuroprotective effect at all concentrations studied: neuronal survival is 51%, 46%, 47%, and 46%, respectively, for 1, 0.1, 0.01, and 0.001 mM.

Thus, neuroprotective activity under the influence of glutamate is shown by all substances. Magnesium comenate shows the greatest, and practically equal, efficiency in all studied concentrations, almost doubling the survival rate of neurons. Less pronounced protective activity is shown by comenic acid, the protective effect of which at a concentration of 1 mM is not inferior to magnesium comenate, but at 0.1 and 0.01 mM it is approximately two times less effective. Magnesium sulfate turns out to be the least effective, and its effect at all concentrations increases the survival of neurons approximately twice less effectively than that of magnesium comenate.

#### 2.2.4. Stress Protective Action In Vivo

**Figure 7:**
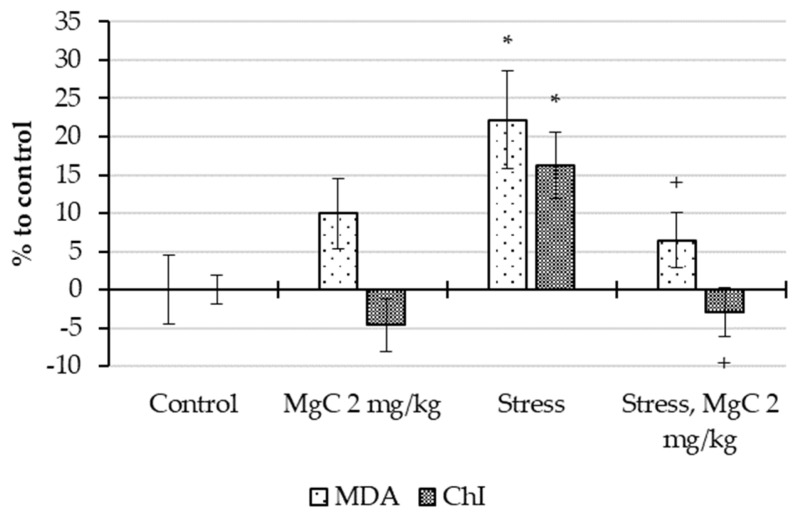
Effect of magnesium comenate on the MDA level and chemiluminescence intensity (ChI) in the brain tissues of rats under stress. *—*p* < 0.05 in relation to control, +—*p* < 0.05 in relation to stress.

In the study of the intensity of oxidative processes in the brain of rats, it is found (Figure 7) that in the absence of stress exposure, MC at a dose of 2 mg/kg increases the MDA content by 10% and reduces the level of chemiluminescence intensity by 5%, but these changes are statistically unreliable. The stress effect leads to an increase in the content of MDA by 22%, and the intensity of chemiluminescence by 16% (*p* < 0.05). The use of MC during the period of stress at a dose of 2 mg/kg prevents the development of oxidative processes, which is expressed by a statistically significant decrease in MDA and the intensity of the chemiluminescent reaction (*p* > 0.05).

**Figure 8:**
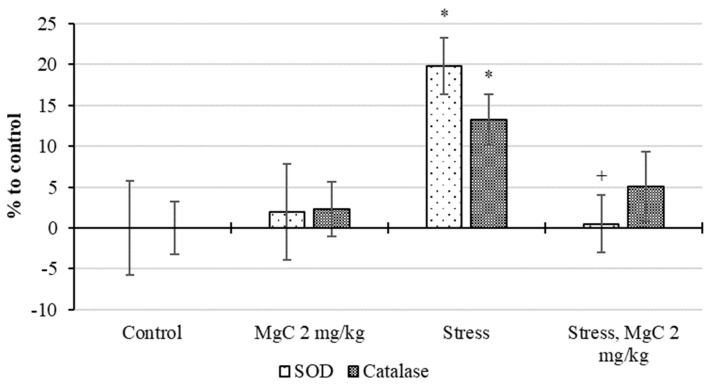
Effect of magnesium comenate on the activity of SOD and catalase in the brain tissues of rats under stress. *—*p* < 0.05 in relation to control, +—*p* < 0.05 in relation to stress.

The study of the antioxidant system parameters show that stress exposure leads to an increase in the activity of SOD and catalase by 20% and 13%, respectively (Figure 8). The use of MC during the period of stress at a dose of 2 mg/kg contributes to maintaining the activity of these enzymes at the level of control values.

**Figure 9:**
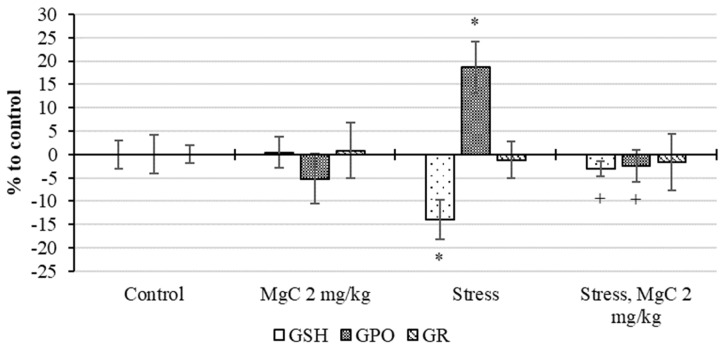
Effect of magnesium comenate on the content of reduced glutathione (GSH), activity of glutathione peroxidase (GPO), and glutathione reductase (GR) in rat brain tissues under stress. *—*p* < 0.05 in relation to control, +—*p* < 0.05 in relation to stress.

Also, stress exposure leads to a decrease in GSH levels by 14% and an increase in glutathione peroxidase activity by 19% (*p* < 0.05). The use of MC at a dose of 2 mg/kg during the period of stress prevents changes in these parameters (Figure 9).

Thus, the use of magnesium comenate during the period of stress prevents the development of oxidative stress in the brain of rats and maintains the parameters of the antioxidant system at the level of the physiological norm.

## 3. Discussion

It is known that the activation of free radical processes is part of the body’s nonspecific response to any external influence, called the general adaptation syndrome or stress [31,32,33,34]. In addition, it is now established that ROS are an integral part of intracellular signaling processes [35,36,37,38,39]. However, due to their high reactivity, ROS, when accumulated excessively, damage the cell by oxidizing proteins, lipids, and DNA. It has now been shown that ROS, reactive nitrogen species, as well as the radicals of biological molecules formed from them, are a key link in many pathologies of the nervous system. In particular, their pathogenetic role has been shown in cerebral stroke, Alzheimer’s disease, Parkinson’s disease, Huntington’s disease, amyotrophic lateral sclerosis, and multiple sclerosis [40,41,42].

In our study, stress exposure leads to an increase in the intensity of free radical oxidation and lipid peroxidation processes in the brain of rats, a decrease in GSH levels, and an increase in the activity of antioxidant enzymes glutathione peroxidase, SOD, and catalase, which indicates the induction of oxidative stress. The use of magnesium comenate leads to the preservation of these parameters at the control level, i.e., at the level of the physiological norm, which is consistent with the results obtained in the CPL model system, in which its antiradical effect is established. Together, the results in vitro and in vivo indicate an increase in the magnesium comenate of the antioxidant resource of the brain and its antioxidant stress-protective effect.

ROS are involved in the functioning of the cytoskeleton, on the work of which, in particular, the functional activity of mitochondria, which provide the energy needs of cells, depends [43]. ROS can regulate cytoskeletal changes at several levels: directly through redox modification of cytoskeletal structural proteins and indirectly through modification of proteins or signaling pathways that regulate cytoskeletal dynamics [44]. Therefore, the cytoskeleton is one of the first to respond to the induction of oxidative stress. Excessive production of free radicals affects the polymerization of both F-actin and microtubules in neurons, leading to destruction of the cytoskeleton [45,46]. It was also found that antioxidant system is involved in the processes of building the cytoskeleton. In particular, it has been shown that disulfide bonds between α- and β-tubulin subunits disrupted by peroxynitrot are restored by the glutaredoxin reductase system, which leads to the normalization of tubulin polymerization [47]. In addition, intracellular signaling pathways can reversibly modulate microtubule polymerization [48]. These data suggest that compounds with antioxidant activity may influence the functioning of the cytoskeleton. In the above experiments, the addition of hydrogen peroxide to cultures of the spinal ganglia results in a strong inhibition of neurite growth. This effect is a consequence of the inhibition of cytoskeleton assembly processes under oxidative stress, as well as other metabolic disorganization processes resulting from oxidative modification of biomolecules. Magnesium comenate significantly prevents the disruption of the growth processes of neurites of the spinal ganglia exposed to hydrogen peroxide. We believe that this effect is associated with its antioxidant activity, established in the CPL model system, as well as in experiments with stress exposure in rats.

Another way to implement the protective effect of magnesium comenate under oxidative stress, as well as to stimulate the growth of neurites under normal conditions, seems to be associated with Na^+^/K^+^-ATPase. Magnesium comenate is a chelate complex of comenic acid and magnesium ions. It is known that chelate complexes with calcium and magnesium ions exhibit neurotrophic properties with the subsequent activation of a specific molecular mechanism of membrane signaling. The neurotrophic action of comenic acid is realized due to the activation of a specific molecular mechanism of membrane signaling, which includes the Na^+^/K^+^-ATPase isoform as an alpha-3 signal transducer. This mechanism is realized during the formation of chelate complexes of comenic acid with magnesium and calcium ions [49]. It is known that Na^+^/K^+^-ATPase in its active state prevents the accumulation of reactive oxygen species in neurons, and, thus, increases their resistance to oxidative stress [50].

We suggest that the main factors of neurite growth of spinal ganglia in normal conditions and under conditions of oxidative stress is the effect of magnesium comenate on ion homeostasis, which ensures the energy stability of neuronal metabolism. Its ability to regulate the transducer function of Na^+^/K^+^-ATPase, which modulates cell growth, plays the role of a neuronal cell oxidative resistance factor, a protector from oxidative stress.

Thus, the effect of magnesium comenate on the growth of neurites of the spinal ganglia is provided in two main ways: the direct antioxidant effect of magnesium comenate, as well as its effect on the functional activity of Na^+^/K^+^-ATPase, which ensures not only the stability of ion homeostasis, but also prevents the accumulation of ROS.

Together with aspartate, glutamate is the main excitatory neurotransmitter in the brain. Glutamate binds and activates ionotropic and metabotropic glutamate receptors. Excess extracellular glutamate can lead to excitotoxicity in vitro and in vivo in acute strokes, such as ischemic stroke, due to the hyperactivation of ionotropic glutamate receptors. In addition, chronic excitotoxicity has been suggested to play a role in numerous neurodegenerative diseases, including amyotrophic lateral sclerosis, Alzheimer’s disease, and Huntington’s disease [51,52]. The pathogenetic cascade triggered by glutamate includes an increasing influx of Ca^2+^ into the cytoplasm; activation of phospholipases, endonucleases, proteases, and nitric oxide synthase; and the formation of harmful free radicals and reactive oxygen and nitrogen species, which leads to the degradation of cellular and extracellular structures [53].

In the study, magnesium comenate shows high neuroprotective activity at all concentrations studied. Comparator substances show different results: comenic acid shows the maximum effect at the highest concentration, and magnesium sulfate shows equal effectiveness at all concentrations studied. Since cell damage by free radicals is an essential part of the excitotoxic effect, we believe that a significant part of the neuroprotective effect of magnesium comenate is due to its antioxidant properties, as well as the effect on Na^+^/K^+^-ATPase, which also prevents the accumulation of ROS.

Another possibility of the neuroprotective action of magnesium comenate is magnesium blockade of NMDA receptors. It is known that Mg^2+^ is a voltage-dependent blocker of NMDA glutamate receptors [54]. By blocking NMDA receptors, magnesium sulfate reduces glutamatergic signaling, which alters calcium influx and leads to reduced excitotoxicity [55,56]. We suggest that one of the possible ways to implement the neuroprotective activity of magnesium comenate may be its effect on the functioning of NMDA glutamate receptor ion channels.

## 4. Materials and Methods

### 4.1. Chemical Part

IR spectra were recorded on a Bruker Vertex 70 IR Fourier spectrometer with an attachment of frustrated total internal reflection on a diamond crystal, spectral resolution was ±4 cm^−1^.

Thermogravimetric analysis was performed on a STA-409 PC Luxx synchronous thermal analyzer (Netzsch, Selb, Germany). The tests were carried out in an oxidizing atmosphere (air) in alundum crucibles under conditions of programmed isothermal heating with an α-Al_2_O_3_ standard at a heating rate of 10 °C/min, temperature range 30–1000 °C.

The elemental composition of the complex was determined by quantitative X-ray fluorescence analysis with energy dispersion. EDX-8000 device (Shimadzu, Tokyo, Japan) based on silicon drift detector (SDD) with thermoelectric cooling. Analysis conditions: an X-ray tube with a rhodium anode was used as a source of exciting radiation, exposure time was 500 s, the diameter of the irradiated zone was 10 mm, the angle of incidence on the sample was 45°, the scattering angle to the detector was 45°, the processes of scattering and reflection of radiation took place in a vacuum environment. To prepare the sample, we used 1.0–1.2 g of the sample ground to 10 µm in an agate mortar. The sample was pressed into a tablet with a diameter of 20 mm (WAX (0.7 g) served as a substrate) using a mold with a force of 15 T.

X-ray diffraction analysis of the crystal of the compound (C_12_H_22_O_18_Mg) was performed on a SuperNova, Dual, Cu at zero, Atlas S2 automatic four-circle diffractometer at 293(2) K. The structure was solved using the Olex2 software packages and SHELXT, and refined using the package SHELXL [30] using the least squares method. The main characteristics of the experiment and the parameters of the unit cell: triclinic syngony, space group P-1, M = 478.60 g/mol, a = 6.7265(2) Å, b = 7.0802(2) Å, c = 10.7367(4) Å, α = 103.337(3)°, β = 96.095(2)°, γ = 103.071(2)°, V = 477.84(3) Å^3^, Z = 1, μ(Cu Kα) = 1.711 mm^−1^, D_calculated_ = 1.663 г/cм^3^, F(000) = 250.0, shooting angle area θ = 8.584–152.2°; reflection index intervals: −8 ≤ h ≤ 8, −8 ≤ k ≤ 8, −12 ≤ l ≤ 13, number of measured reflections 9635 (8.584° ≤ 2ϴ ≤ 152.2°), number of independent reflections 1990 (R_int_ = 0.0180, R_sigma_ = 0.0104). R-factors [I > 2σ(I)]: R_1_ = 0.0248 (wR_2_ = 0.0657), R-factors for all reflections: R_1_ = 0.0251 (wR_2_ = 0.0657); GOOF пo F^2^ 1.107, Δρ_max_/Δρ_min_ = 0.38/−0.21 e Å^−3^. The XRD results for the compound were deposited with the Cambridge Crystallographic Data Center (Cambridge, UK) (CCDC no. 2207835).

Electronic spectra were recorded on a U-2900 double-beam spectrophotometer (Hitachi, Tokyo, Japan) in quartz cuvettes (l = 10 mm) in the spectral range 190–400 nm. Empirical spectra were smoothed using the fast Fourier transform (FFT) using the OriginLab 2019 (version 2019) software package.

NMR spectra were recorded on a JNM-ECA 400 pulse spectrometer (JEOL, Tokyo, Japan) [400 (^1^H) and 101 MHz (^13^C)]. Standard conditions (298 K), solution of the substance in D_2_O. TMS and residual solvent signals were used as a standard. ^1^H NMR spectra in the range −2–12 ppm, ^13^C NMR spectra in the range −20–220 ppm. The ACDLABS v.10_0 NMRMAN software (version 10_0) was used to process the results.

The purity of the obtained compounds and the course of the reaction were monitored by TLC on Sorbfil PTSKh-AF-A plates (Russia), eluent acetone–hexane 1:1, developer—iodine vapor, UV detector. For the synthesis, Mg(CH_3_COO)_2_·4H_2_O (analytical grade, >99.5%, Moscow, Russia) was used as starting compounds. All experiments were performed using bi-distilled water.

**Scheme 1:**
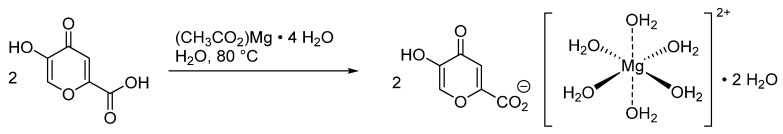
Synthesis of magnesium comenate.

Magnesium comenate [Mg(HCom)_2_(H_2_O)_6_]·2H_2_O was obtained by reacting comenic acid with magnesium acetate hydrate when heated in water (Scheme 1). The structure of the reaction product was studied in detail using elemental X-ray phase analysis, thermal analysis, IR-Fourier spectrometry, UV spectroscopy, NMR spectroscopy, and X-ray diffraction analysis.

#### 4.1.1. 5-Hydroxy-4-oxo-4H-pyran-2-carboxylic (comenoic) Acid

Comenic acid was obtained with a yield of 80% by oxidation of glucose using Gluconobacter oxydans strain 003 according to known methods [57,58] followed by purification by column chromatography. IR spectrum (ν, cm^−1^): 3339 (O8-H H_2_L), 3089 (C-H), 2997–2467 (O-H H_2_O), 1726 (C4=O7), 1628 (C=C), 1599 (C1=O), 1420 (C5-O), 1219 (C1-O), 1204 (C5-O-H).

NMR spectrum ^1^H [D_2_O, 298 K], (δ, ppm): 7.03 c (1H, C3-H), 8.02 c (1H, C6-H).

NMR spectrum ^13^C [D_2_O, 298 K], (δ, ppm): 176.9 (C4), 164.2 (COOH), 156.5 (C2), 146.4 (C5), 142.3 (C6), 115.3 (C3).

#### 4.1.2. Magnesium 5-Hydroxy-4-oxo-4H-pyran-2-carboxylate (comenate)

Magnesium comenate was synthesized according to the following procedure: a solution of comenic acid (1.00 g, 6.4 mmol) in 25 mL of water at a temperature of 80 ± 2 °C was mixed with a solution of magnesium acetate (0.69 g Mg(CH_3_COO)_2_·4H_2_O, 3.2 mmol). As a result, the reaction mass acquires a pH value of 4.5–5.0 and turns yellow. Magnesium comenate was isolated from the solution by evaporation by about 3/4 of the original volume, while magnesium comenate began to crystallize from the hot solution. The resulting product was further purified by recrystallization from bi-distilled water. Yield 2.61 g (85%).

IR spectrum (v, cm^−1^): (O8-H H_2_L), 3190, 3093 (C-H), 2976 (O-H H_2_O), 1691 ν(C4=O), 1601 ν_as_(COO^–^), 1556 (C1=O), 1462, 1352 ν_s_(COO^–^), 1271, 1213 (C1-O), 1157 (C5-O-H), 1101, 935, 893, 854, 804, 771, 663, 561, 517, 411.

^1^H NMR spectrum [D_2_O, 298 K], (δ, ppm): 7.03 s. (1H, C3-H), 8.02 s. (1H, at C6-H).

^13^C NMR spectrum [D_2_O, 298 K], (δ, ppm): 176.91 (C4), 164.15 (C1), 156.48 (C2), 146.43 (C5), 142.26 (C6), 115.34 (C3).

Elemental composition of the C_12_H_22_O_18_Mg presented in Table 4.

### 4.2. Biological Part

#### 4.2.1. Chemicals and Supplements

Chemicals and additives were purchased from Sigma-Aldrich (St. Louis, MO, USA): propidium iodide, HEPES, poly-L-lysine, thiobarbituric acid; from PanEco (Moscow, Russia): minimum essential medium, fetal calf serum, glutamine, salts for solutions, and culture media. Comenic acid and magnesium comenate, used in the work, were synthesized in the Department of Biologically Active Substances (Kuban State University).

The antioxidant properties of magnesium comenate in vitro were studied using a model system that generates free radicals—the “citrate-phosphate-luminol” (CPL) system of the following composition: 4 mL of phosphate buffer (105 mM KCl, 20 mM KH_2_PO_4_, 4 mM sodium citrate; pH = 7.45) with the addition of luminol (10 mM). The formation of reactive oxygen species (ROS) was initiated by introducing 30 μL of a 35 mM solution of iron sulfate with constant stirring. Registration of CL was carried out with a Lum-100 device (Russia) for 5 min. The results of the experiments were determined by the intensity of chemiluminescence (in c.u.) and calculated as a percentage of the control. Control measurements were carried out without adding the test substances. The final concentration of the substance in the cuvette was 0.1 mg/mL and 0.01 mg/mL. The intensity of free radical chemiluminescence of the CPL model system (control) is taken as 100% [20]. Statistical analysis of the results was performed using Student’s *t*-test in Statistica 10.

#### 4.2.2. Obtaining Cultures of the Spinal Ganglia

The spinal ganglia of chicken embryos were cultivated according to the previously described methods [59] using a nutrient medium of the following composition: fetal calf serum—30%; medium needle (DMEM)—55%; Hank’s solution—12%; glucose solution (40%)—2%; solution of L-glutamine (0.2 M)—1%.

#### 4.2.3. Modeling Oxidative Stress

Oxidative stress (OS) was induced 24 h after the start of spinal ganglia cultivation by adding hydrogen peroxide at a final concentration of 10 mM to the nutrient medium for 30 min. After 30 min, the medium with hydrogen peroxide was removed, the cultures were placed in a fresh culture medium, a solution of magnesium comenate at a final concentration of 1 mM to 0.0001 mM, comenic acid (1–0.01 mM), or magnesium sulfate (1–0.01 mM). Some cultures (control) were not exposed to hydrogen peroxide and test substances.

#### 4.2.4. Accounting for Neurite Growth

Quantitative analysis of growth rates of neurites in the culture of spinal ganglia of chick embryos was performed 48 h after the start of cultivation on an inverted microscope Invertoscopes ID 03 (Oberkochen, Germany) by intravital microscopy in phase contrast. The following parameters were determined in relative units:

- The maximum value of the growth zone (MGZS), equal to the distance from the edge of the explant to the tip of the longest neurite;
- The number of neurites (NN), which was determined by counting the number of processes in a segment of 200 μm at a distance of 250 μm from the edge of the ganglion;
- Growth intensity (GI), defined as the product of MGZS and the density of neurites, which was determined by grading NN on a scale: less than 10 neurites—1; 10–30—2; 30–50—3; more than 50—4.

Statistical analysis of the results was performed using the Kruskal–Wallis test and Mann–Whitney U-test in Statistica 10 (version 10). Data are presented as M ± SE. Differences were considered significant at *p* < 0.05.

#### 4.2.5. Obtaining Cultures of Cerebellar Neurons

For experiments with cultures of neurons, we used 7–8 day old cultures of cerebellar neurons obtained by the method of enzymatic–mechanical dissociation from the brain of 7–9 day old Wistar rats as described in article [6] with some modifications. After sacrificing the pups with ethyl ether, they were treated with 70% ethyl alcohol. After that, the brain was removed and placed on the horizontal surface of the Maximov chamber. Next, the brain was washed with Ca^2+^- and Mg^2+^-free PBS and the cerebellum was removed. Then the cerebellum was transferred into the well of the Maximov chamber containing 2–3 mL of PBS and excised with a scalpel. The tissue were exposed for 20 min to trypsin (0.05%) and EDTA (0.02%) dissolved in PBS solution at 36.5 °C. After that, the tissue was washed three times with PBS r and once in the culture medium and then subjected to mechanical dissociation in the culture medium, of the following composition: 90% minimum essential medium, 10% fetal calf serum, 2 mM glutamine, 10 mM HEPES buffer, and 5 mM KCl. The cell suspension was centrifuged at 1500 rpm for 1 min. After that, the supernatant was removed, and the precipitate was resuspended in the nutrition medium with 25 mM KCl necessary for survival of the cerebellum granular neurons. The cultures were grown in 96-well plates coated with poly-L-lysine. Each well was added with 0.1 mL cell suspension at final density of 3–5 × 10^3^ cell/mm^2^. The cells were cultured in a CO_2_ incubator at 36.5 °C and 98% relative humidity.

#### 4.2.6. Modeling of Excitotoxic Effects

Exposure to glutamate (100 μM) was carried out in a balanced salt solution (SSR) of the following composition (mM): NaCl—154, KCl—25, Na_2_HPO_4_—0.35, CaCl_2_—2.3, NaHCO_3_—3.6, glucose—5, 6, HEPES—5 (pH 7.5). The duration of exposure was 10 min, control cultures were placed for 10 min in SSR without glutamate. After that, the cultures were returned to the original culture medium and placed in a CO_2_ incubator for 4.5 h. Comenate magnesium, comenic acid, and magnesium sulfate were added to cultures immediately after their return to the culture medium in final concentrations from 1 to 0.001 mM.

#### 4.2.7. Determining the Level of Neuron Death

The level of neuron death was determined by the following method. The cultures were preliminarily fixed with a special solution (alcohol 70%, formalin 20%, glacial acetic acid 10%) and stained with trypan blue. The first method consisted of the morphological analysis of cultures on an inverted microscope ID 03, which took into account the number of living and dead neurons in 3–5 fields of view. The results were determined by the number of intact neurons as a percentage. Statistical analysis of the results was performed using the two-way analysis of variance and Mann–Whitney U-test in Statistica 10. Data are presented as M ± SE. Differences were considered significant at *p* < 0.05.

#### 4.2.8. Experiments on Laboratory Animals

The antioxidant activity of magnesium comenate was studied in a combined stress model in rats.

Wistar rats were used in our work. The animals were kept in a vivarium with free access to food and water. During the experiment, the animals were kept in standard conditions, with free access to water and food, in TECNIPLAST type IV plastic cages, with 3–4 rats in one cage (in accordance with the rules of animal accommodation). The conditions of the animals were standardized: temperature: 20 ± 3 °C, humidity: 48 ± 2%, lighting mode: day/night (from 6.00 a.m. until 6.00 p.m./from 6.00 p.m. until 6.00 a.m.). Birch chips were used as bedding. During the whole experiment, the animals consumed standard concentrated mixed grain feed according to state standard GOST R 50258. The experiments were carried out in accordance with the requirements of the “Guide for the Care and Use of Laboratory Animals” [60], European Community Directives 2010/63/EU and “Guide for working with laboratory animals, including the ethical principles of animal testing (3R principle) of the V.M. Gorbatov Federal Research Center for Food Systems of the Russian Academy of Sciences”. The study was approved by the bioethical commission of the V.M. Gorbatov Federal Research Center for Food Systems of the Russian Academy of Sciences (Protocol No. 4/2021 on 15 April 2021).

The experiments were carried out on 48 male Wistar rats weighing 240–250 g. The rats were subjected to immobilization–cold exposure daily for three days. Immobilization–cold stress was modeled by placing animals in a case at a temperature of 7 ± 1 °C. On the fourth day of the experiment, under general anesthesia with Zoletil 100 (Virbac, France), the animals were decapitated. The brain was removed and placed in liquid nitrogen until the moment of biochemical analysis. Magnesium comenate was orally administered to rats daily for 3 days at a dose of 2 mg/kg before stress and during stress.

The following groups of male rats were formed:

1. Control;
2. Magnesium comenate;
3. Stress;
4. Stress + magnesium supplement.

#### 4.2.9. Determination of the Pro-Oxidant and Antioxidant Status of the Brain

The intensity of oxidative processes in rat brain tissues was determined by the chemiluminescent method [61] on a Lum 100 instrument (Moscow, Russia). The results of the experiments were evaluated by the intensity of chemiluminescence in c.u. The content of malondialdehyde (MDA) was determined spectrophotometrically by the amount of the colored product obtained by interaction with thiobarbituric acid at a wavelength of 532 nm [62]. The activity of superoxide dismutase was determined by the inhibition of the rate of reduction of nitroblue tetrazolium in the non-enzymatic system of phenazine methasulfate and NADH. Catalase activity was determined by a method based on the ability of hydrogen peroxide to form a stable colored complex with ammonium molybdate [63]. Enzyme activity was expressed as a percentage.

The level of reduced glutathione was determined at a wavelength of 412 nm by the amount of a colored product formed upon interaction with Ellman’s reagent. The activity of glutathione peroxidase was assessed by the ability of the enzyme to oxidize the reduced glutathione contained in the supernatant [64].

Statistical analysis of the results was performed using the Kruskal–Wallis test and Mann–Whitney U-test in Statistica 10. Data are presented as M ± SE. Differences were considered significant at *p* < 0.05.

## 5. Conclusions

Thus, an individual chemical compound was obtained, its crystal structure was characterized, and the empirical formula was established. The obtained data expand the spectrum of the characterized substances containing magnesium ions in their composition. Studies of the pharmacological activity of magnesium comenate indicate its antioxidant, stress- and neuro-protective effects. The implementation of these effects occurs both due to the direct antioxidant action and due to the effect on Na^+^/K^+^-ATPase, the activation of which also contributes to the reduction in oxidative damage, and also, probably, due to the influence on the functioning of NMDA receptor ion channels. The lack of experimental models of specific neurodegenerative diseases can be attributed to the limiting factors of this work. On the other hand, we have shown the possible neuro-protector activity of magnesium comenate. We suggest that the presented data indicate the expediency of further studies of magnesium comenate, as a compound potentially applicable in medicine for the pharmacological correction of conditions associated with oxidative and excitotoxic damage to nerve cells. The task of future research should include a detailed study of the mechanisms of the neuro-protective action of magnesium comenate, as well as its effectiveness in relation to specific pathologies.

## Figures and Tables

**Table 1:** Experimental data on thermal analysis of the complex compound of the Mg^2+^ with comenic acid.

Temperature Range, °C	Thermal Effect, °C	Effect Type	Mass Loss, %	The Amount Consumed/ Released Energy, mW/mg	Processes that Occur in a Substance When Heated
25–205	137.8	endo	29.73	−4.193	Dehydration and the beginning of the ligand decomposition process
205–285	254.6	exo	11.83	–	Removal of hydroxyl and carboxyl groups of the ligand
285–304	276.2	endo	3.94	−1.122	
304–400	365.1	exo	26.10	+12.94	Continued decomposition of two ligand molecules and combustion of decomposition products
400–575	473.9	exo	19.51	+6.403	
575–998.9	–	–	0.09	–	End of ligand decomposition and combustion of decay products
Residual mass—8.79% (998.9 °C)					

**Table 2:** Comparison of physical and chemical analysis data to establish the gross formula of magnesium comenate.

	Experimentally Calculated Mass Fraction, %			Estimated Mass Fraction, %
	Thermogravimetry	XRF Elemental Analysis	Complexometric Titration	
Mg	5.30 ± 0.05	5.14 ± 0.02	5.25 ± 0.03	5.08
HCom^−^	61.4 ± 0.6	63.52 ± 0.25	–	64.81
H_2_O	29.7 ± 0.3	–	–	30.11

**Table 3:** The level of reduction in free radicals in the model CPL system in the presence of comenic acid, magnesium comenate (% of control).

Substance	Substance Concentration, mg/mL	
	0.01	0.1
Magnesium comenate	30.67 ± 1.20 *	67.03 ± 0.51 *#
Comenic acid	33.29 ± 1.36 *	69.39 ± 1.16 *#

Note: *—*p* < 0.001 compared to control; #—*p* < 0.001 vs. 0.01 mg/kg.

**Table 4:** Elemental composition C_12_H_22_O_18_Mg.

	C	H	O	Mg
**Found, %**	28.11	4.45	57.81	5.14
**Calculated, %**	30.11	4.63	60.17	5.08

## Data Availability

Not applicable.

## Supplementary Materials

The following supporting information can be downloaded at: https://www.mdpi.com/article/10.3390/ijms24098046/s1.

## References

[1] Garre-Olmo J. (2018). Epidemiologia de la enfermedad de Alzheimer y otras demencias. Rev. Neurol..

[2] Hugo J., Ganguli M. (2014). Dementia and cognitive impairment: Epidemiology, diagnosis, and treatment. Clin. Geriatr. Med..

[3] Graham N.S., Sharp D.J. (2019). Understanding neurodegeneration after traumatic brain injury: From mechanisms to clinical trials in dementia. J. Neurol. Neurosurg. Psychiatry.

[4] Cobley J.N., Fiorello M.L., Bailey D.M. (2018). 13 reasons why the brain is susceptible to oxidative stress. Redox Biol..

[5] Singh A., Kukreti R., Saso L., Kukreti S. (2019). Oxidative Stress: A Key Modulator in Neurodegenerative Diseases. Molecules.

[6] Kravtsov A., Kozin S., Basov A., Butina E., Baryshev M., Malyshko V., Moiseev A., Elkina A., Dzhimak S. (2022). Reduction of deuterium level supports resistance of neurons to glucose deprivation and hypoxia: Study in cultures of neurons and on animals. Molecules.

[7] Kozin S., Skrebitsky V., Kondratenko R., Kravtsov A., Butina E., Moiseev A., Malyshko V., Baryshev M., Elkina A., Dzhimak S. (2021). Electrophysiological Activity and Survival Rate of Rats Nervous Tissue Cells Depends on D/H Isotopic Composition of Medium. Molecules.

[8] Morén C., de Souza R.M., Giraldo D.M., Uff C. (2022). Antioxidant Therapeutic Strategies in Neurodegenerative Diseases. Int. J. Mol. Sci..

[9] Veronese N., Zurlo A., Solmi M., Luchini C., Trevisan C., Bano G., Manzato E., Sergi G., Rylander R. (2016). Magnesium Status in Alzheimer’s Disease: A Systematic Review. Am. J. Alzheimer’s Dis. Other Dement..

[10] Maier J.A., Castiglioni S., Locatelli L., Zocchi M., Mazur A. (2021). Magnesium and inflammation: Advances and perspectives. Semin. Cell Dev. Biol..

[11] Yamanaka R., Shindo Y., Oka K. (2019). Magnesium Is a Key Player in Neuronal Maturation and Neuropathology. Int. J. Mol. Sci..

[12] Romeo V., Cazzaniga A., Maier J.A.M. (2019). Magnesium and the blood-brain barrier in vitro: Effects on permeability and magnesium transport. Magnes. Res..

[13] Maier J.A.M., Locatelli L., Fedele G., Cazzaniga A., Mazur A. (2022). Magnesium and the Brain: A Focus on Neuroinflammation and Neurodegeneration. Int. J. Mol. Sci..

[14] Pickering G., Mazur A., Trousselard M., Bienkowski P., Yaltsewa N., Amessou M., Noah L., Pouteau E. (2020). Magnesium Status and Stress: The Vicious Circle Concept Revisited. Nutrients.

[15] Mathew A.A., Panonnummal R. (2021). ‘Magnesium’-the master cation-as a drug-possibilities and evidences. Biometals.

[16] Xu Z.P., Li L., Bao J., Wang Z.H., Zeng J., Liu E.J., Li X.G., Huang R.X., Gao D., Li M.Z. (2014). Magnesium protects cognitive functions and synaptic plasticity in streptozotocin-induced sporadic Alzheimer’s model. PLoS ONE.

[17] Brookfield K.F., Vinson A. (2019). Magnesium sulfate use for fetal neuroprotection. Curr. Opin. Obstet. Gynecol..

[18] Ortiz J.F., Ruxmohan S., Saxena A., Morillo Cox Á., Bashir F., Tambo W., Ghani M.R., Moya G., Córdova I. (2020). Minocycline and Magnesium As Neuroprotective Agents for Ischemic Stroke: A Systematic Review. Cureus.

[19] Kravtsov A.A., Shurygin A.Y., Skorokhod N.S., Khaspekov L.G. (2011). Neuroprotector effect of comenic acid against cytotoxic action of glutamate in vitro in cultured neurons of lead-poisoned rat pups. Bull. Exp. Biol. Med..

[20] Kozin S.V., Kravtsov A.A., Kravchenko S.V., Ivashchenko L.I. (2021). Cytoprotective and Antioxidant Effects of Meconic Acid in Model Systems. Bull. Exp. Biol. Med..

[21] Shurygina L.V., Zlishcheva E.I., Khablyuk V.V., Kravtsova A.N., Abramova N.O., Zlishcheva L.I., Kravtsov A.A. (2015). Comparative Analysis of Antioxidant Properties of Comenic Acid and Potassium Comenate in Modeled Immobilization Stress. Bull. Exp. Biol. Med..

[22] Demina N.I., Zlishcheva L.I., Shurygin A. (1998). Exposure to baliz-2 and some of its components on structural changes in the epidermis during healing of a full thickness ear wound in the rabbit. Dokl. Akad. Nauk..

[23] Chumasov E.I., Shurygin A.I., Soldatova S., Svetikova K.M., Drobyshevski A.I. (1993). Nerve regeneration under the influence of baliz-2 and laktovit. Morfologiia.

[24] Rogachevskii I.V., Plakhova V.B., Penniyaynen V.A., Terekhin S.G., Podzorova S.A., Krylov B.V. (2022). New approaches to the design of analgesic medicinal substances. Can. J. Physiol. Pharmacol..

[25] Shurygina L.V., Zlishcheva E.I., Kravtsov A.A. (2018). Neurotrophic Action of Comenic Acid and Its Derivatives Potassium Comenate and Calcium Comenate. Bull. Exp. Biol. Med..

[26] Shurygina L.V., Zlishcheva E.I., Kravtsova A.N., Kravtsov A.A. (2017). Antioxidant and Antiamnestic Effects of Potassium Comenate and Comenic Acid under Conditions of Normobaric Hypoxia with Hypercapnia. Bull. Exp. Biol. Med..

[27] Kondratenko R.V., Chepkova A.N., Shurygin A.Y., Skrebitskii V.G. (2003). Comenic acid prevents post-stress enhancement of long-term potentiation in rat hippocampus. Bull. Exp. Biol. Med..

[28] Ismailova G.M., Iminova I.M., Tulaganov A.A. (2002). Synthesis of Mg(II) Complexes with Nicotinic Acid and Nicodine. Pharm. Chem. J..

[29] Case D.R., Zubieta J., Gonzalez R., Doyle R.P. (2021). Synthesis and Chemical and Biological Evaluation of a Glycine Tripeptide Chelate of Magnesium. Molecules.

[30] Sheldrick G.M. (2015). Crystal structure refinement with SHELXL. Acta Crystallogr. C Struct. Chem..

[31] Feelisch M., Cortese-Krott M.M., Santolini J., Wootton S.A., Jackson A.A. (2022). Systems redox biology in health and disease. EXCLI J..

[32] Basov A.A., Kozin S.V., Bikov I.M., Popov K.A., Moiseev A.V., Elkina A.A., Dzhimak S.S. (2019). Changes in Prooxidant-Antioxidant System Indices in the Blood and Brain of Rats with Modelled Acute Hypoxia which Consumed a Deuterium-Depleted Drinking Diet. Biol. Bull..

[33] Basov A., Fedulova L., Baryshev M., Dzhimak S. (2019). Deuterium-depleted water influence on the isotope ^2^H/^1^H regulation in body and individual adaptation. Nutrients.

[34] Tekutskaya E.E., Ryabova I.S., Kozin S.V., Popov K.A., Malyshko V.V. (2022). Changes in Free Radical Processes under the Influence of Low-Frequency Electromagnetic Field in Rats. Bull. Exp. Biol. Med..

[35] Noctor G., Foyer C.H. (2016). Intracellular Redox Compartmentation and ROS-Related Communication in Regulation and Signaling. Plant Physiol..

[36] Zhang J., Wang X., Vikash V., Ye Q., Wu D., Liu Y., Dong W. (2016). ROS and ROS-Mediated Cellular Signaling. Oxid. Med. Cell. Longev..

[37] Zhang B., Pan C., Feng C., Yan C., Yu Y., Chen Z., Guo C., Wang X. (2022). Role of mitochondrial reactive oxygen species in homeostasis regulation. Redox Rep..

[38] Morris G., Gevezova M., Sarafian V., Maes M. (2022). Redox regulation of the immune response. Cell. Mol. Immunol..

[39] Resende R., Fernandes T., Pereira A.C., Marques A.P., Pereira C.F. (2022). Endoplasmic Reticulum-Mitochondria Contacts Modulate Reactive Oxygen Species-Mediated Signaling and Oxidative Stress in Brain Disorders: The Key Role of Sigma-1 Receptor. Antioxid. Redox Signal..

[40] Bjorklund G., Zou L., Peana M., Chasapis C.T., Hangan T., Lu J., Maes M. (2022). The Role of the Thioredoxin System in Brain Diseases. Antioxidants.

[41] Liu Z., Zhou T., Ziegler A.C., Dimitrion P., Zuo L. (2017). Oxidative Stress in Neurodegenerative Diseases: From Molecular Mechanisms to Clinical Applications. Oxid. Med. Cell. Longev..

[42] Liu X., Henty-Ridilla J.L. (2022). Multiple roles for the cytoskeleton in ALS. Exp. Neurol..

[43] Alberti P., Semperboni S., Cavaletti G., Scuteri A. (2022). Neurons: The Interplay between Cytoskeleton, Ion Channels/Transporters and Mitochondria. Cells.

[44] Oswald M.C.W., Garnham N., Sweeney S.T., Landgraf M. (2018). Regulation of neuronal development and function by ROS. FEBS Lett..

[45] Allani P.K., Sum T., Bhansali S.G., Mukherjee S.K., Sonee M. (2004). A comparative study of the effect of oxidative stress on the cytoskeleton in human cortical neurons. Toxicol. Appl. Pharmacol..

[46] Chalimeswamy A., Yogananda Thanuja M., Ranganath S.H., Pandya K., Kompella U.B., Srinivas S.P. (2022). Oxidative Stress Induces a Breakdown of the Cytoskeleton and Tight Junctions of the Corneal Endothelial Cells. J. Ocul. Pharmacol. Ther..

[47] Landino L.M., Moynihan K.L., Todd J.V., Kennett K.L. (2004). Modulation of the redox state of tubulin by the glutathione/glutaredoxin reductase system. Biochem. Biophys. Res. Commun..

[48] Wilson C., González-Billault C. (2015). Regulation of cytoskeletal dynamics by redox signaling and oxidative stress: Implications for neuronal development and trafficking. Front. Cell. Neurosci..

[49] Lopatina E.V., Karetsky A.V., Krylov B.V. (2009). Regenerative Anti-Inflammatory Agent and Methods of Treatment Using This Agent. RU Patent.

[50] Boldyrev A., Bulygina E., Yuneva M., Schoner W. (2003). Na/K-ATPase regulates intracellular ROS level in cerebellum neurons. Ann. N. Y. Acad. Sci..

[51] Lewerenz J., Maher P. (2015). Chronic Glutamate Toxicity in Neurodegenerative Diseases-What is the Evidence?. Front. Neurosci..

[52] Verma M., Lizama B.N., Chu C.T. (2022). Excitotoxicity, calcium and mitochondria: A triad in synaptic neurodegeneration. Transl. Neurodegener..

[53] Pregnolato S., Chakkarapani E., Isles A.R., Luyt K. (2019). Glutamate Transport and Preterm Brain Injury. Front. Physiol..

[54] Nikolaev M.V., Magazanik L.G., Tikhonov D.B. (2012). Influence of external magnesium ions on the NMDA receptor channel block by different types of organic cations. Neuropharmacology.

[55] Elsayed N.A., Boyer T.M., Burd I. (2021). Fetal Neuroprotective Strategies: Therapeutic Agents and Their Underlying Synaptic Pathways. Front. Synaptic Neurosci..

[56] Bachnas M.A., Akbar M.I.A., Dachlan E.G., Dekker G. (2021). The role of magnesium sulfate (MgSO_4_) in fetal neuroprotection. J. Matern. Fetal Neonatal Med..

[57] Shurygin A.Y., Shurygina L.V., Lobova N.N. (2012). Method for Producing Comenic Acid. RU Patent.

[58] Shurygin A.Y. (2006). The Bacterial Strain Gluconobacter Oxydans-03 Is a Balise Producer and a Method for Producing Balise. RU Patent.

[59] Kravtsov A.A., Shurygin A.Y., Shurygina L.V., Zlishcheva L.I., Abramova N.O., Khaspekov L.G. (2009). Prenatal action of lead acetate on the antioxidant glutathione system of the brain of newborn rats in vivo and on neurite growth in vitro. Neurochem. J..

[60] National Research Council (US) Committee for the Update of the Guide for the Care and Use of Laboratory Animals (2011). Guide for the Care and Use of Laboratory Animals.

[61] Kozin S.V., Kravtsov A.A., Elkina A.A., Zlishcheva E.I., Shurygina L.V., Baryshev M.G., Barysheva E.V., Moiseev A.V. (2019). Isotope Exchange of Deuterium for Protium in Rat Brain Tissues Changes Brain Tolerance to Hypoxia. Biophysics.

[62] Kravtsov A.A., Kozin S.V., Elkina A.A., Shashkov D.I., Baryshev M.G., Vasilevskaya E.R., Fedulova L.V., Popov K.A., Malyshko V.V., Moiseev A.V. (2018). Effect of drinking ration with reduced deuterium content on brain tissue prooxidant-antioxidant balance in rats with acute hypoxia model. J. Pharm. Nutr. Sci..

[63] Kozin S.V., Kravtsov A.A., Kravchenko S.V., Ivashchenko L.I. (2021). Antioxidant and anxiolytic effect of Bifidobacterium adolescentis and Lactobacillus acidophilus under conditions of normobaric hypoxia with hypercapnia. Vopr. Pitan.

[64] Shurygina L.V., Kravtsov A.A., Zlishcheva E.I., Khaspekov L.G. (2017). The in vitro and in vivo neuroprotective activity of sodium comenate in stress. Neurochem. J..

