# Supplementary material for: Study of the Magnesium Comenate Structure, Its Neuroprotective and Stress-Protective Activity
Source: Int J Mol Sci. 2023 Apr 28;24(9):8046. doi: 10.3390/ijms24098046 (PMC10178379; doi:10.3390/ijms24098046)
Supplement: Supplementary file 1 [file ijms-24-08046-s001.zip › ijms-2279223-supplementary.pdf]

Table S1. Crystallographic data, experimental parameters, and refinement of the magnesium comenate structure (according to X-ray diffraction data)

Parameter	Value
Gross formula	$C_{12}H_{22}MgO_{18}$
M,g/mol	478.60
Temperature, *K*	293(2)
Crystallographic system	triclinic
Space group	P-1
a, Å	6.7265(2)
b, Å	7.0802(2)
c, Å	10.7367(4)
α, deg.	103.337(3)
β, deg.	96.095(2)
γ, deg.	103.071(2)
V, $Å^3$	477.84(3)
Z	1
$\varrho_{(calc.)}$, g/cm^3	1.663
μ, mm^{-1}	1.711
F(000)	250.0
Crystal size, mm	0.417 × 0.324 × 0.307
Radiation	Cu K*α* (*λ* = 1.54184)
Data collection range 2Θ, deg	8.584-152.2
Ranges *h*, *k*, *l*	$-8 \le h \le 8$, $-8 \le k \le 8$, $-12 \le l \le 13$
Number of measured reflections	9635
Number of independent reflections (R_{int}, R_σ)	1990 (0.0180, 0.0104)
Data/Restrictions/Parameters	1990/0/179
GOOF by F^2	1.107
R- factor (I>2σ(I))	$R_1 = 0.0248$, $wR_2 = 0.0655$
R- factor (all data)	$R_1 = 0.0251$, $wR_2 = 0.0657$
$\Delta\varrho_{max}/\Delta\varrho_{min}$, e $Å^{-3}$	0.38/-0.21

Table S2. **Main bond lengths for [Mg(HCom)$_2$(H$_2$O)$_6$]·2H$_2$O**

**Bond**	***d*, Å**	**Bond**	***d*, Å**
Mg(1)–O(2^1)	2.0684(8)	O(8)–C(5)	1.3481(13)
Mg(1)–O(2)	2.0684(7)	O(7)–C(4)	1.2498(13)
Mg(1)–O(1^1)	2.0841(8)	O(4)–C(1)	1.2508(13)
Mg(1)–O(1)	2.0841(8)	C(5)–C(4)	1.4512(14)
Mg(1)–O(3)	2.0400(8)	C(5)–C(6)	1.3519(15)
Mg(1)–O(3^1)	2.0400(8)	C(4)–C(3)	1.4403(14)
O(6)–C(2)	1.3467(12)	C(2)–C(1)	1.5230(14)
O(6)–C(6)	1.3583(13)	C(2)–C(3)	1.3510(15)
O(5)–C(1)	1.2463(13)		

Note: ^{1}-X,1-Y,2-Z

Table S3. **Main values of bond angles for [Mg(HCom)$_2$(H$_2$O)$_6$]·2H$_2$O**

**Angle**	**ω, degree**	**Angle**	**ω, degree**
O(2)Mg(1)O(2^1)	180.0	C(2)O(6)C(6)	118.94(8)
O(2^1)Mg(1)O(1^1)	90.83(3)	O(8)C(5)C(4)	120.80(9)
O(2)Mg(1)O(1)	90.83(3)	O(8)C(5)C(6)	119.27(9)
O(2^1)Mg(1)O(1)	89.17(3)	C(6)C(5)C(4)	119.90(10)
O(2)Mg(1)O(1^1)	89.17(3)	O(7)C(4)C(5)	121.06(9)
O(1^1)Mg(1)O(1)	180.00(3)	O(7)C(4)C(3)	124.32(9)
O(3^1)Mg(1)O(2^1)	86.85(3)	C(3)C(4)C(5)	114.60(9)
O(3)Mg(1)O(2)	86.85(3)	O(6)C(2)C(1)	112.80(9)
O(3^1)Mg(1)O(2)	93.15(3)	O(6)C(2)C(3)	122.42(9)
O(3)Mg(1)O(2^1)	93.15(3)	C(3)C(2)C(1)	124.77(9)
O(3^1)Mg(1)O(1^1)	90.32(3)	C(5)C(6)O(6)	122.96(9)
O(3)Mg(1)O(1)	90.32(3)	O(5)C(1)O(4)	127.81(10)
O(3)Mg(1)O(1^1)	89.68(3)	O(5)C(1)C(2)	115.19(9)
O(3^1)Mg(1)O(1)	89.68(3)	O(4)C(1)C(2)	116.99(9)
O(3^1)Mg(1)O(3)	180.0	C(2)C(3)C(4)	121.06(10)

Note: ^{1}-X,1-Y,2-Z

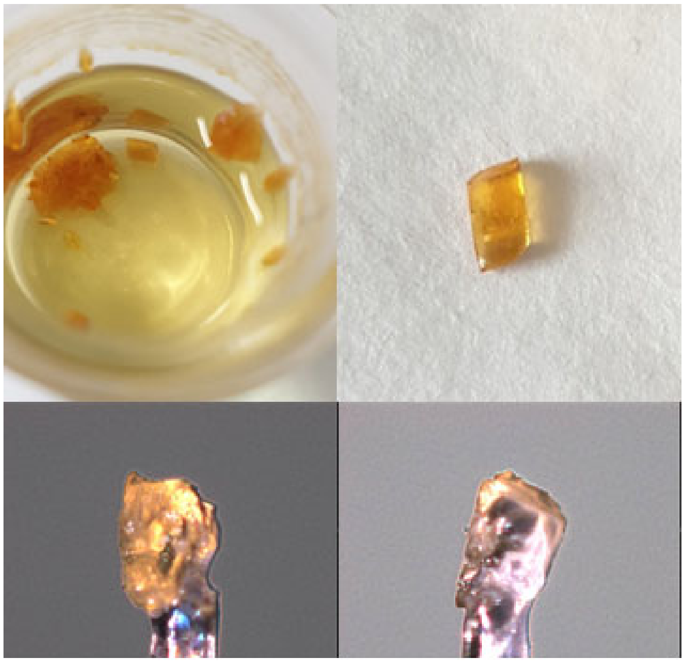

Figure S1. Several single crystals were obtained, the size of which is suitable for X-ray diffraction studies.
